# The Roles of Solute Carriers in Auditory Function

**DOI:** 10.3389/fgene.2022.823049

**Published:** 2022-01-26

**Authors:** Fuping Qian, Xiaoge Jiang, Renjie Chai, Dong Liu

**Affiliations:** ^1^ School of Life Sciences, Nantong University, Nantong, China; ^2^ Department of Rehabilitation Medicine, The Second People’s Hospital of Nantong, Affiliated Rehabilitation Hospital of Nantong University, Nantong, China; ^3^ State Key Laboratory of Bioelectronics, Jiangsu Province High-Tech Key Laboratory for Bio-Medical Research, Department of Otolaryngology Head and Neck Surgery, Zhongda Hospital, Southeast University, Nanjing, China; ^4^ Co-Innovation Center of Neuroregeneration, Nantong University, Nantong, China; ^5^ Institute for Stem Cell and Regeneration, Chinese Academy of Science, Beijing, China; ^6^ Beijing Key Laboratory of Neural Regeneration and Repair, Capital Medical University, Beijing, China; ^7^ Department of Otolaryngology Head and Neck Surgery, Sichuan Provincial People’s Hospital, University of Electronic Science and Technology of China, Chengdu, China

**Keywords:** solute carrier, SLC, transporter, hereditary hearing loss, deafness gene

## Abstract

Solute carriers (SLCs) are important transmembrane transporters with members organized into 65 families. They play crucial roles in transporting many important molecules, such as ions and some metabolites, across the membrane, maintaining cellular homeostasis. SLCs also play important roles in hearing. It has been found that mutations in some SLC members are associated with hearing loss. In this review, we summarize SLC family genes related with hearing dysfunction to reveal the vital roles of these transporters in auditory function. This summary could help us understand the auditory physiology and the mechanisms of hearing loss and further guide future studies of deafness gene identification.

## Introduction

**FIGURE 1:**
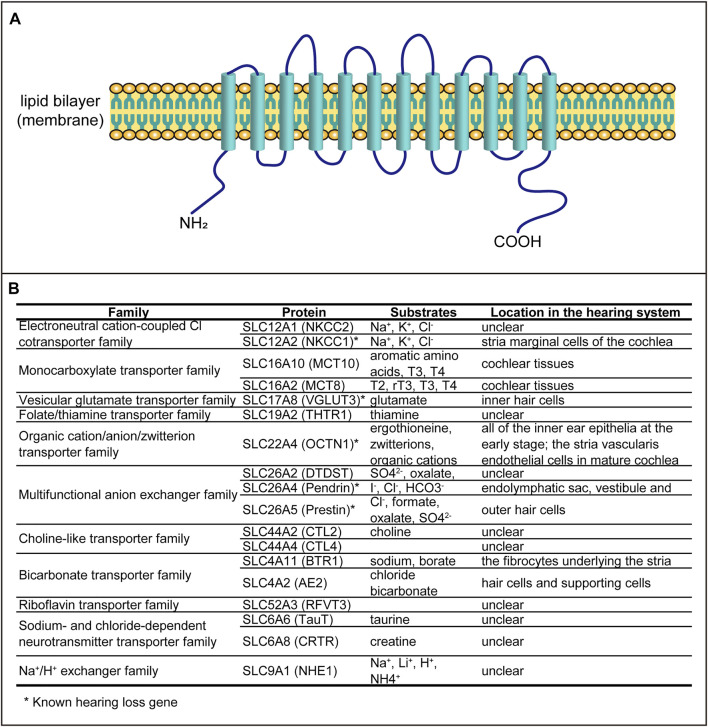
SLC proteins involved in auditory function. **(A)** Schematic structure of the transporter containing 12 transmembrane domains. **(B)** All SLC proteins associated with hearing dysfunction.

SLCs are a large family of transporters and play vital roles in transporting many molecules, such as amino acids, glucose, ions, fatty acids, and neurotransmitters. There are 65 families of SLCs (SLC1–65), with more than 400 members (http://slc.bioparadigms.org/). Most of the SLC proteins have 12 transmembrane domains (Figure 1A) and have been found in many tissues. These SLCs play multiple roles in cellular ion homeostasis, cellular metabolism, and cell survival.

It has been reported that some of the SLCs are associated with hearing loss, including auditory organ development and hearing dysfunction. In this review, we summarize all SLCs related to hearing systematically (Figure 1B) in order to explore the expression patterns and possible functions.

Hearing is one of the most important sensory functions, and hearing loss would cause great inconvenience to the daily life of deaf people. The causes of hearing loss vary, from congenital to acquired impairments, but the defects of genes account for the majority. More than 200 hearing loss genes, including the syndromic and the nonsyndromic hearing loss genes, were identified in the last decades (https://hereditaryhearingloss.org/). Among these genes, five of them belong to the SLC family. Besides, another 13 SLC members were reported to be associated with hearing loss or involved in auditory organ development. These SLC genes, although most of them act as transporters, have different expression patterns in auditory organs and distinct function in hearing.

## SLC12A2

**FIGURE 2:**
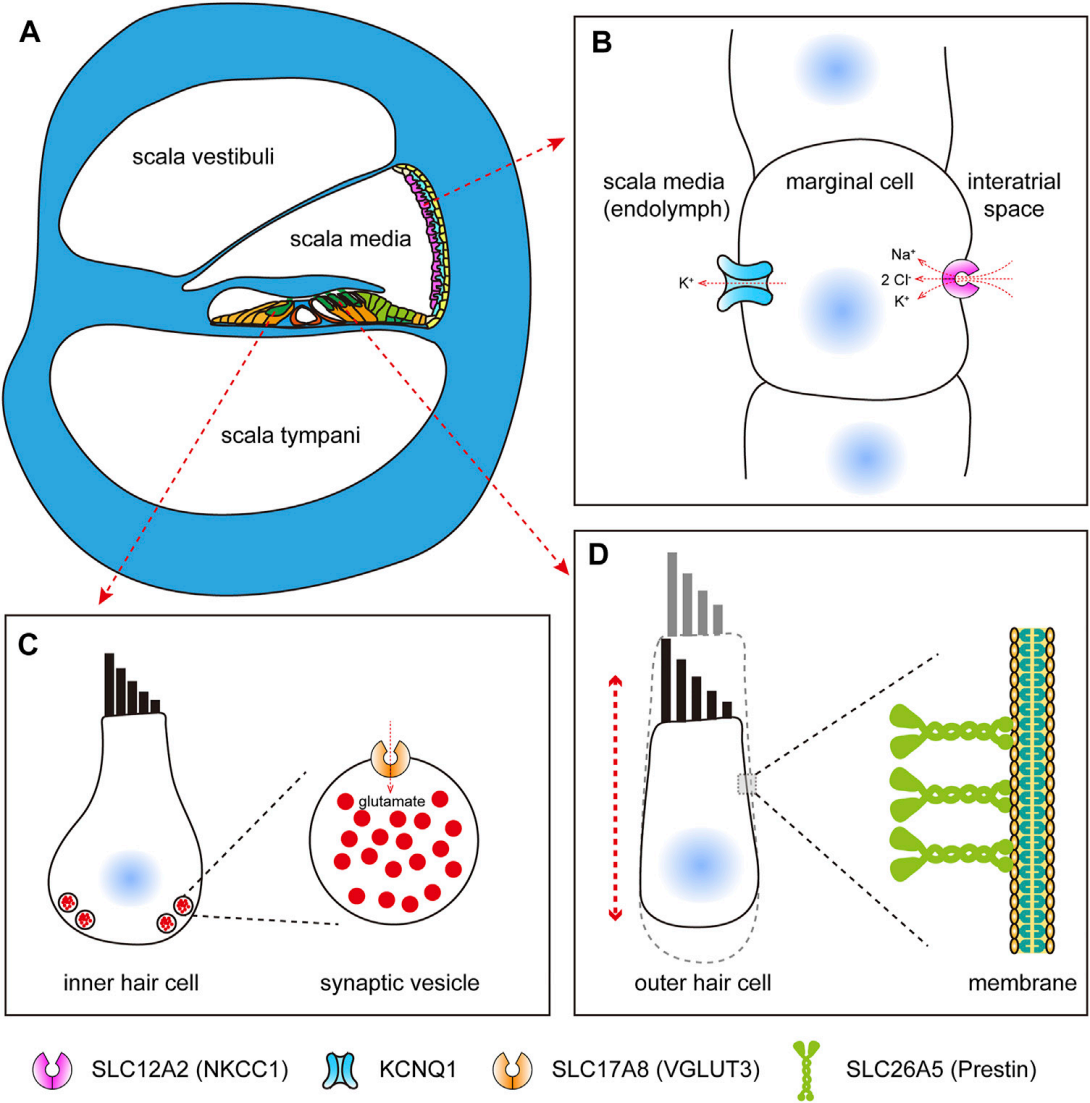
SLC proteins expressed in mammalian cochlear cells. **(A)** Schematic diagram of mammalian cochlea (cross section). **(B)** Schematic diagram of SLC12A2, which functions in potassium recycling in stria marginal cells, maintaining the endocochlear potential. **(C)** Schematic diagram of SLC17A8, which functions in transporting glutamate in inner hair cells. **(D)** Schematic diagram of SLC26A5, which acts as motor proteins, driving somatic electromotility in outer hair cells.

The solute carrier family 12 (SLC12) gene, which encodes electroneutral cation-coupled chloride cotransporters, is very important in some physiological processes, such as cell volume regulation, modulation of intraneuronal chloride concentration, transepithelial ion movement, and blood pressure regulation (Arroyo et al., 2013). There are nine members in this family, namely, *SLC12A1* to *SLC12A9*, and some members have been reported to be associated with human diseases. As is known, the mammalian cochlea is the auditory organ that is essential for hearing (Figure 2A). The *solute carrier family 12 member 2* (*SLC12A2*) gene, encoding the Na–K–2Cl cotransporter-1 (NKCC1), is mainly expressed in the stria marginal cell of the cochlea (Crouch et al., 1997; Goto et al., 1997; Mizuta et al., 1997), which is critical for the maintenance of endocochlear potential because of its role in potassium recycling, keeping the endolymph at a high potassium concentration (Figure 2B). In mice, cochlear NKCC1 mRNA and protein decrease with increasing age (Liu et al., 2014), and knockout of the *Slc12a2* gene results in complete collapse of Reissner’s membrane, and the *Slc12a2* ^*−/−*^ mice are deaf and exhibit classic shaker/waltzer behavior (Delpire et al., 1999). Besides, mutation in *SLC12A2* also leads to sensorineural hearing loss in humans (Macnamara et al., 2019; McNeill et al., 2020; Mutai et al., 2020). Therefore, the loss of NKCC1 would be an important factor that causes age-related hearing loss (ARHL). It has been proved that aldosterone can enhance NKCC1 protein expression by increasing protein stability (Ding et al., 2014; Bazard et al., 2020), which provides a potential therapeutic for ARHL.

## SLC17A8

Hair cells in the mammalian cochlea detect mechanical signals from the tectorial membrane and transmit them to the auditory neurons by releasing the transmitters into the synaptic cleft, which are then captured by the postsynaptic receptors. Glutamate, as the most abundant neurotransmitter in the central nervous system, plays a key role in the auditory function. Vesicular glutamate transporter 3 (VGLUT3), encoded by the *slc17a8* gene, is exclusively expressed in hair cells and localized to the basal end of hair cells (Figure 2C), and the mutant *slc17a8* hair cells showed reduced ribbon-associated synaptic vesicles and absent postsynaptic action currents in zebrafish (Obholzer et al., 2008). Similarly, in the cochlea of mice, the *Slc17a8* gene is expressed in the inner hair cells, but not in the outer hair cells, and mice with *Slc17a8* deletion lack auditory nerve responses to acoustic stimuli (Ruel et al., 2008; Seal et al., 2008), and also, the glutamate transmission deficit results in sensorineural deafness because of a mutation of *SLC17A8* in humans (Ruel et al., 2008). In addition, more and more mutations within the *SLC17A8* gene were identified in different families with hearing loss (Ryu et al., 2016; Ryu et al., 2017). A recent study has showed that tinnitus caused by sodium salicylate treatment was also due to the disruption of VGLUT3 in cochlear inner hair cells (Zhang et al., 2020).

## SLC22A4

*SLC22A4*, also named as OCTN1, a 551-amino acid–long protein, is a pH-dependent organic cation transporter (Tamai et al., 1997) and has a broad expression in many organs or tissues, such as the colon (Peltekova et al., 2004; Meier et al., 2007), mammary glands (Lamhonwah et al., 2011), and airways (Horvath et al., 2007). It functions as an exchanger which carries organic cations or zwitterions across the plasma membrane through sodium-dependent or independent manners, and the substrates of this transporter include tetraethylammonium (TEA) (Yabuuchi et al., 1999), ergothioneine (ET) (Grundemann et al., 2005), and so on. The *SLC22A4* gene was identified as a susceptibility gene for rheumatoid arthritis, and it was negatively regulated by RUNX1, a transcription factor which was also significantly associated with rheumatoid arthritis (Tokuhiro et al., 2003). In addition, the expression of *SLC22A4* was regulated by nuclear factor-κB (NF-κB) and inflammatory cytokines, such as interleukin-1β (IL-1β) and tumor necrosis factor-α (TNF-α) (Maeda et al., 2007). It was also reported that *SLC22A4* deficiency increased the susceptibility to Crohn’s disease (Peltekova et al., 2004; Newman et al., 2005). Mice with *Slc22a4* gene knockout exhibited greater susceptibility to intestinal inflammation under the ischemia and reperfusion model (Kato et al., 2010). All of the results demonstrated the important roles of *SLC22A4* in chronic inflammation.

In recent years, the mutation of *SLC22A4* was found to be associated with hereditary hearing loss in humans. In mammalian auditory organs, *SLC22A4* is expressed in all of the inner ear epithelia at the early stage, including the hair cells and spiral ganglion neurons; however, the expression is restricted at the apical surface of stria vascularis (SV) endothelial cells in the mature cochlea (Ben Said et al., 2016), and the mutation of the *SLC22A4* gene causes autosomal recessive nonsyndromic hearing loss, DFNB60, in humans (Ben Said et al., 2016; Chiereghin et al., 2021). However, the detailed physiological function of *SLC22A4* in the hearing process and the underlying mechanisms of hearing loss caused by *SLC22A4* variants are still unknown.

## SLC26A4

The *solute carrier family 26 member 4* (*SLC26A4* or *PDS*) gene, encoding the protein pendrin, is the causal gene of Pendred syndrome, which is a recessively inherited disorder with hearing loss as the obvious feature (Everett et al., 1997; Li et al., 1998). In addition, mutation of the *SLC26A4* gene is also associated with the enlargement of the vestibular aqueduct syndrome (EVAS) (Maciaszczyk and Lewiński, 2008). It was also reported that pendrin may regulate blood pressure because patients with *SLC26A4* mutation are likely to be resistant to high blood pressure (Kim et al., 2017).

The *SLC26A4* gene was found to be expressed in the thyroid at high levels, and it can help the thyroid follicular cells transport iodine (Everett et al., 1997; Maciaszczyk and Lewiński, 2008). In the inner ear, the *SLC26A4* gene was detected in the endolymphatic sac, vestibule, and cochlea (Yoshino et al., 2006). However, pendrin may play different roles in the cochlear and vestibular systems because gene therapy of the *Slc26a4* gene mutation restored the hearing phenotype but not the vestibular function in mice (Kim et al., 2019).

The protein pendrin contains 12 transmembrane domains, and it functions in sodium-dependent transportation of anions, such as iodides, chlorides, and bicarbonates (Dawson and Markovich, 2005; Maciaszczyk and Lewiński, 2008).

## SLC26A5

The SLC16A5 (also named as prestin) protein, encoded by the *solute carrier family 26 member 5* (*SLC26A5*) gene, is the most well-studied solute carrier in hearing-related research. In the mammalian inner ear, it is specifically expressed in the basolateral membrane of outer hair cells (Figure 2D), and deficiency of *SLC26A5* results in nonsyndromic hearing loss (Liu et al., 2003). In nonmammalian vertebrates and insects, the homolog of *SLC26A5* was also reported to be expressed in the auditory organs (Weber et al., 2003). The protein prestin is more than 700 amino acids in length, and nearly the full length of the protein is required for its proper expression and normal function (Zheng et al., 2005). Unlike most members of solute carrier family 26 (SLC26), which transport different anion substrates across the membrane, mammalian SLC26A5 functions as voltage-dependent motor proteins that drive somatic electromotility in outer hair cells (Zheng et al., 2000), which was thought to be crucial for frequency selectivity and sensitivity of mammalian hearing (Liberman et al., 2002; Liu et al., 2003; Cheatham et al., 2004; Dallos et al., 2008). Indeed, as for prestin, the motor function is an innovation of therians and is concurrent with diminished transporter capabilities (Tan et al., 2011). Nonmammalian prestin acts as an anion transporter; however, in mammals, prestin functions as both a motor protein (Zheng et al., 2000) and a weak transporter (Mistrík et al., 2012). Prestin can form higher order oligomers (Zheng et al., 2006) and interact with the cystic fibrosis transmembrane conductance regulator (CFTR) for activation (Homma et al., 2010). Moreover, it can be functionally regulated by calcium/calmodulin (Keller et al., 2014). Recently, the structure-based mechanism of prestin electromotive signal amplification was illustrated (Ge et al., 2021), providing a better understanding of the molecular basis of hearing and a crucial guidance for the treatment of hearing impairment.

## Other SLCs

Except for the SLC members discussed above, which have been identified as hearing loss genes, some other SLCs, waiting to be verified as deafness genes, were reported to function in hearing-related processes.

### SLC4A2 and SLC4A11

SLC4 family members are bicarbonate transporters and play vital roles in acid–base homeostasis (Romero et al., 2013). Among the 10 members (SLC4A1–5 and SLC4A7–11), two genes were reported to function in the hearing process.

The *SLC4A2* gene, encoding HCO_3_^−^/Cl^−^ anion exchangers, was reported to be expressed in mammalian inner ear cells, including hair cells and supporting cells (Stanković et al., 1997; Hosoya et al., 2016), and the gene mutant mice were virtually deaf (Gawenis et al., 2004). In our previous study, *slc4a2b*, the homolog of human *SLC4A2*, was proven to be required for hair cell development and function in zebrafish (Qian et al., 2020).

*SLC4A11* gene mutation causes genetic corneal dystrophies. However, in addition to corneal disease, deficiency of this gene also leads to sensorineural deafness (Desir et al., 2007; Groger et al., 2010; Vilas et al., 2013). In the inner ear, the *SLC4A2* gene is expressed in the fibrocytes underlying the stria vascularis, and *SLC4A2*-null fibrocytes manifest intracellular vacuolations and extracellular edemas, which cause reduced endocochlear potential and hearing threshold (Groger et al., 2010).

### SLC6A6 and SLC6A8

SLC6 is a sodium- and chloride-dependent neurotransmitter transporter family, and it has more than 20 members, namely, SLC6A1–21 (Pramod et al., 2013). The substrates of these transporters include serotonin, dopamine, norepinephrine, GABA, taurine, creatine, and some amino acids. The SLC6 family genes are important for normal biological and physiological processes and related to a number of human diseases.

The *SLC6A6* gene encodes the taurine transporter, and mice with *Slc6a6* gene knockout develop multisystemic dysfunctions, including hearing impairment caused by loss of hair cells and spiral ganglion neurons (Warskulat et al., 2007). However, a homozygous *SLC6A6* mutation in two boys with early-onset retinal degeneration did not cause hearing loss (Preising et al., 2019).

Another SLC6 family member, *SLC6A8*, encoding the creatine transporter, was also found to be associated with hearing loss. It was reported that a patient with double deletion of the *SLC6A8* and *BAP31* genes suffered from severe dystonia and sensorineural deafness (Osaka et al., 2012).

### SLC9A1

The SLC9 family is mainly characterized by Na^+^/H^+^ exchangers (Donowitz et al., 2013). So far, the gene reported to be related to hearing loss in this family is *SLC9A1*, encoding Na^+^/H^+^ exchanger 1 (NHE1), which is important in maintaining intracellular pH homeostasis by exchanging one intracellular H^+^ for one extracellular Na^+^(Fliegel, 2009). Complete or near-complete loss of function of *SLC9A1* causes the Lichtenstein–Knorr syndrome, which is characterized by cerebellar ataxia and sensorineural hearing loss (Guissart et al., 2015). However, deafness may not be an essential phenotypic feature of *SLC9A1* mutation because other patients with variant *SLC9A1* did not show hearing loss (Iwama et al., 2018).

### SLC12A1

SLC12 is an electroneutral cation–coupled chloride cotransporter family (Arroyo et al., 2013). Except the known deafness gene *SLC12A2* discussed earlier, disrupted *SLC12A1* was also reported to be involved in hearing loss. In that case, translocation of the *SLC12A1* and *ATE1* (arginyltransferase 1) genes was found in a boy with nonsyndromic hearing loss; however, no hearing impairment occurred in his brother, father, and grandfather who have the same translocation (Vona et al., 2014). In another case, a homozygous missense mutation within the *SLC12A1* gene caused type I antenatal Bartter syndrome (ABS), without hearing deficits (Halperin et al., 2019). All of these demonstrate that *SLC12A1* has a role in hearing loss, but it is probably through polygenic or multifactorial ways.

### SLC16A2 and SLC16A10

SLC16 family members are mainly responsible for the transport of monocarboxylates, such as lactate and pyruvate; therefore, they are called monocarboxylate transporters (MCTs) (Halestrap, 2013). Distinct with other members in this family, *SLC16A2* (also named as MCT8) and *SLC16A10* (also named as MCT10), which share similarities with each other, prefer to transport the iodothyronines (T4 and T3) (Friesema et al., 2008). As known to us, the thyroid hormone is required for hair cell survival and normal hearing (Rüsch et al., 2001; Mustapha et al., 2009; Ng et al., 2015). Unsurprisingly, *SLC16A2* and *SLC16A10* are expressed in the cochlear tissues (Sharlin et al., 2011), and they were also reported to have a role in the maintenance of cochlear hair cells and hearing through T3-dependent mechanisms (Sharlin et al., 2018).

### SLC19A2

SLC19 is a folate/thiamine transporter family, and there are three members (SLC19A1–3), of which *SLC19A1* transports folates but not thiamine, and the other two transport thiamine but not folates (Zhao and Goldman, 2013). Mutations in the *SLC19A2* gene, encoding thiamine transporter 1 (THTR1), were reported to be associated with thiamine-responsive megaloblastic anemia (TRMA) (Scharfe et al., 2000; Ozdemir et al., 2002; Ghaemi et al., 2013; Setoodeh et al., 2013; Sun et al., 2018; Amr et al., 2019), which is characterized by early-onset diabetes mellitus, anemia, and sensorineural deafness.

### SLC26A2

The SLC26 family genes encode multifunctional anion exchangers and anion channels, and 11 members (SLC26A1–11) are included in this family (Alper and Sharma, 2013). In addition to the two members, *SLC26A4* and *SLC26A5*, discussed above, another gene, *SLC26A2*, encoding for a sulfate/chloride transporter, may be associated with hearing loss. In zebrafish, the *slc26a2* gene was proven to be critical for otic development and hair cell survival, and loss of function of *slc26a2* led to defective auditory organ development and impaired hearing (Liu et al., 2015).

### SLC44A2 and SLC44A4

The SLC44 family, with five members (SLC44A1–5), is a choline-like transporter family (Traiffort et al., 2013).

SLC44A2, also named choline transporter–like protein 2 (CTL2), is a transmembrane glycoprotein. It was identified as the target of the Kresge Hearing Research Institute-3 (KHRI-3) antibody, which can lead to autoimmune hearing loss by binding to *SLC44A2* and blocking its transporter function (Nair, 2004). Moreover, knockout of the *Slc44a2* gene also caused hair cell and spiral ganglion neuron loss, especially in the basal turn of the cochlea, and *Slc44a2* null mice exhibited high-frequency hearing loss (Kommareddi et al., 2015).

*SLC44A4* encodes the choline transport protein CTL4. Mutations in this gene were found in a Chinese family with postlingual nonsyndromic mid-frequency sensorineural hearing loss, and knockdown of the *slc44a4* gene in zebrafish led to significant defects in the otic vesicle and lateral line neuromast development, accompanied by defective hearing (Ma et al., 2017). Further evidence showed that *SLC44A4* mutation disrupted its choline uptake function and acetylcholine synthesis ability, leading to hearing loss.

### SLC52A3

Brown–Vialetto–Van Laere (BVVL) syndrome is a rare neurodegenerative disease characterized by sensorineural hearing loss and a variety of cranial nerve palsies (Sathasivam, 2008; Yonezawa and Inui, 2013). Mutations in the *SLC52A3* gene, encoding riboflavin transporter 3 (RFVT3), was found in the BVVL syndrome (Johnson et al., 2010; Bosch et al., 2011), and a high dose of riboflavin improved the syndrome (Anand et al., 2012), indicating that mutation of the *SLC52A3* gene might be a cause of BVVL syndrome. However, the mechanisms by which *SLC52A3* functions in the auditory system remain unclear.

## Conclusion

According to the World Health Organization (WHO), one in four, about 2.5 billion, people worldwide will be living with some degree of hearing loss by 2050, and nearly 60% of hearing loss is caused by genetic factors. Therefore, hereditary hearing loss is a serious problem that needs attention. In the last decades, more than 200 human hearing loss genes have been identified by scientists all over the world.

The SLC family genes, with more than 400 members, encode different types of transporters that function in solute transporting. Deficiency of some of these genes would cause human diseases. Until now, 18 members, as listed in this review, in the SLC family were reported to be associated with the occurrence of hereditary deafness, including five known hearing loss genes, which indicated the crucial roles of solute carriers in hearing function. These SLCs have different expression patterns in the inner ear and have distinct function, and each of them is essential for normal hearing. Mutations in any of these genes would result in inner ear cell dysfunction or even cell death, leading to hearing impairment or hearing loss.

In this review, we systematically summarized all of the SLCs involved in hearing, from their expression to function to the underlying mechanisms. This study would be helpful in understanding the roles of solute transporting for normal hearing, and it can also provide ideas for deafness gene identification.
